# Unsaturated Long-Chain Fatty Acids Activate Resident Macrophages and Stem Cells in a Human Skeletal Muscle Tissue Model

**DOI:** 10.3390/biology12081111

**Published:** 2023-08-09

**Authors:** Xiaoying Chen, Dandan Hao, Nils Becker, Aline Müller, Miguel Pishnamaz, Leo Cornelius Bollheimer, Frank Hildebrand, Mahtab Nourbakhsh

**Affiliations:** 1 Clinic for Geriatric Medicine, RWTH Aachen University Hospital, 52074 Aachen, Germany; xchen@ukaachen.de (X.C.); dand.hao@outlook.com (D.H.); almueller@ukaachen.de (A.M.); cbollheimer@ukaachen.de (L.C.B.); 2 Clinic for Orthopedics, Trauma, and Reconstructive Surgery, RWTH Aachen University Hospital, 52074 Aachen, Germany; nibecker@ukaachen.de (N.B.); mpishnamaz@ukaachen.de (M.P.); fhildebrand@ukaachen.de (F.H.)

**Keywords:** human, skeletal muscle, tissue model, macrophages, stem cells, satellite cells, myoblasts, fatty acids, chemokines, cytokines, aging, sarcopenia

## Abstract

**Simple Summary:**

The ability of the skeletal muscle tissue to regenerate declines with age, leading to the loss of muscle mass, loss of mobility, and severe morbidities in older adults. Animal studies have implied that muscle tissue-resident macrophages and stem cells are important in stimulating the regeneration of aging muscle tissue via metabolic stimuli, such as fatty acids (FAs). However, an ultimate confirmation of these findings in human models was still lacking. Previously, we described an optimal procedure for maintaining human skeletal muscle tissue under experimental conditions for 11 days. Here, we used this procedure to study the abundance and the response of macrophages and stem cells to saturated or unsaturated long-chain fatty acid (FA) species in skeletal muscle tissue specimens from surgeries under experimental conditions. The data showed that only unsaturated long-chain FAs can stimulate the relevant phenotypes of macrophages and stem cells capable of skeletal muscle tissue regeneration. Thus, the outcomes of our study are useful for understanding and improving skeletal muscle tissue regeneration.

**Abstract:**

Phenotypically heterogeneous populations of tissue-resident macrophages and stem cells play important roles in the regeneration of the skeletal muscle tissue. Previous studies using animal and cell culture models implied a beneficial effect of fatty acid (FA) species on tissue regeneration. Here, we applied a human experimental model using excised muscle tissues from reconstructive surgeries to study the effects of FAs on resident macrophages and stem cells in the natural environment of human skeletal muscle tissue. Muscle tissue samples from 20 donors were included in this study. The expression of 34 cytokines/chemokines was determined, using multiplex protein analysis. The phenotypes of macrophages and stem cells were determined immunohistochemically. The numbers of CD80^+^ macrophages correlated with the expression levels of IL-1α, IL-1RA, IL-8, IL-17A, and MCP-1, while the PAX7^+^ and MyoD^+^ stem cell counts were positively correlated with the expression level of CXCL12α, a recognized chemoattractant for muscle stem cells. Treatment of additional tissue sections with FAs revealed that CD80^+^ or MARCO^+^ macrophages- and PAX7^+^ or MyoD^+^ stem cells were simultaneously increased by unsaturated long-chain FAs. Taken together, this is the first experimental demonstration of a coordinated activation of macrophages and stem cells in human skeletal muscle tissue.

## 1. Introduction

The regenerative capacity of human skeletal muscle tissue declines with age, manifesting in a gradual loss of skeletal muscle mass and function in older adults, which is associated with a considerable risk of physical disability, poor quality of life, and death. Obesity, and changes in body composition, such as a redistribution of muscle and fat tissues, an increase in the visceral fat proportion, or a decrease in the subcutaneous fat proportion, can further enhance the progress of skeletal muscle mass loss [1]. Recent studies have suggested that age-related loss of muscle mass is mainly due to changes in the skeletal muscle metabolism, which directly affect energy utilization, fat deposition, and regenerative dysfunction [2].

The coordinated process of skeletal muscle regeneration relies on multiple strictly regulated steps, including muscle stem cell activation, proliferation, and differentiation into myoblasts, as well as myoblast maturation and fusion to form muscle fibers. Thus, the skeletal muscle tissue maintains a homeostatic pool of stem cells and myoblasts that express discrete sets of functional proteins. The characterization of human skeletal muscle stem cells is still the subject of extensive studies. At present, paired box protein 7 (PAX7) and myoblast determination protein 1 (MyoD) are frequently used to detect muscle stem cells and myoblasts, respectively. PAX7 is one of the main regulators of satellite cell function, as it controls the expression of genes enhancing satellite cell survival and proliferation [3]. MyoD is expressed at an early stage of myogenic differentiation, and induces the expression of other genes that facilitate myoblast differentiation and fusion [3].

In addition, the native skeletal muscle tissue comprises other types of cells, such as adipocytes and macrophages, that can directly affect the function and proliferation of myogenic cells at different levels [4]. The importance of tissue-resident macrophages in the regulation of tissue homeostasis and inflammation has only recently emerged. They are a heterogeneous population of prenatally established cells that are maintained and developed in tissue, independent of hematopoiesis and hematopoietic stem cells [5]. The characterization of tissue macrophages mostly relies on marker proteins established in macrophage precursor cells (monocytes) isolated from blood and artificially differentiated in vitro. Further studies using bone-marrow-derived macrophages have led to a general classification of two main phenotypes, namely, proinflammatory (M1) and anti-inflammatory (M2) macrophages, in humans and other species [6]. These experimental guidelines were initially used to study tissue-resident macrophages in animal experiments, or to capture the steady state in human specimens. However, studies within the last decade have established that most tissue-resident macrophage populations in mice and humans develop in the embryonic stage, and are maintained independently to bone-marrow-derived monocytes [7]. Moreover, each tissue has a specific population of macrophages that significantly varies between the two species. Despite many interspecies differences, mice are the preferred model for studying tissue-resident macrophages.

Muscle-tissue-resident macrophages in mice are involved in amplifying both the inflammatory and regenerative processes [6]. Proinflammatory macrophages have been found to promote the proliferation and growth of muscle stem cells, and to inhibit their fusion, whereas anti-inflammatory macrophages can induce differentiation and fusion in stem cells, to form multinucleated myotubes. The sequential regulation of macrophage inflammatory states is critical to muscle regeneration [8]. Thus, the disruption of the inflammatory process may lead to impaired muscle regeneration. Animal studies suggest that the prolonged activation of M1 macrophages can exacerbate muscle tissue damage, while the premature recruitment of M2 macrophages can interfere with regeneration, impede muscle repair, and promote fibrosis [9]. However, macrophages may exhibit a greater functional heterogeneity [10].

Obesity leads to an excessive lipid accumulation in the adipose tissue, and an increased ectopic deposition of lipids in muscle or other tissues. This results in the release of free fatty acids (FAs) that can differentially cause local inflammation [11]. Previously, we reported that saturated and unsaturated long-chain FAs (C16 or C18) differentially activate the expression of multiple cytokine/chemokine genes in human primary skeletal muscle myoblasts [12]. Previous in vitro experiments have demonstrated that FAs are important metabolic mediators of macrophage activation [13]. Although conflicting reports exist, the skeletal muscle tissue of older adults may accumulate a lower number of proinflammatory macrophages than that of younger adults [14,15]. Thus, investigating the effects of FA species on the polarization of skeletal-muscle-tissue-resident macrophages may help in finding a new strategy for the reactivation of skeletal muscle regeneration in older adults.

Previously, we established an in vitro model of human skeletal muscle tissue using surgical specimens [16]. Here, we used this model to compare the effects of different FA species on muscle-tissue-resident macrophages and stem cells in 20 donors of different ages.

## 2. Materials and Methods

### 2.1. Tissue Specimens

Human skeletal muscle samples were obtained from 20 individuals who were undergoing reconstructive surgery at RWTH Aachen University Hospital in Aachen, Germany, after April 2021. Ethical approval (code number EK206/09) was obtained from the local Medical Ethics Committee of RWTH Aachen in November 2009. Informed consent was obtained from all participants involved in this study before the surgeries.

### 2.2. Multiplex Protein Quantification

Sample preparation and analysis were performed using Luminex-xMAP-technology-based Magpix (Thermo Fisher Scientific, Waltham, MA, USA), according to the manufacturer’s instructions. Briefly, skeletal muscle tissue extracts were prepared using Procarta Plex Cell Lysis Buffer (Thermo Fisher Scientific). Total protein concentrations were determined using a Pierce 660 nm Protein Assay Kit (22662, Thermo Fisher Scientific), according to the manufacturer’s instructions. Human Cytokine/Chemokine 34-ProcartaPlex Immunoassays (EPXR340-12167-901, Thermo Fisher Scientific) were used to quantify the expression of 34 human cytokines/chemokines (GM-CSF, MIP-1α, IL-10, IFNγ, IL-1α, IL-1RA, IL-4, IL-6, IL-8, IL-15, IL-17A, IL-21, MCP-1, RANTES, IL-22, CXCL12α, Eotaxin, GRO-α, IFN α, MIP-1 α, TNF-β, IL-1β, IL-2, IL-5, IL-7, IL-9, IL-13, IL-18, IL-23, IL-27, IL-31, IP-10, MIP-1β, and TNF-α) in all protein samples (12.5–25 µL, equivalent to 2.5 mg of tissue). All quantification runs were repeated four times independently, and the mean values of the detected concentrations of each cytokine/chemokine were used for statistical analysis.

### 2.3. Maintenance of, and FA Stimulation in, Human Skeletal Muscle Tissue

Participants’ tissue samples were dissected into contiguous tissue blocks of approximately 18 mm^3^. One block was preserved in 4% formaldehyde (Otto Fischar GmbH, Saarbrucken, Germany) at a temperature of 4 °C immediately after surgery for reference (designated as day 0). The remaining blocks were separately embedded in 1% ultrapure polysaccharide polymer low-melt agarose (Carl Roth GmbH, Karlsruhe, Germany) medium containing DMEM (Biological industries, Kibbutz Beit-Haemek, Israel), 10% fetal bovine serum (PAN Biotech GmbH, Aidenbach, Germany), 100 U/mL of penicillin, and 100 U/mL of streptomycin (PAN Biotech GmbH, Aidenbach, Germany), respectively. For stimulation, analytical-grade fatty acids C16[1]c, C18[2]c, C16, and C18 (all from Biotrend Chemikalien GmbH, Cologne, Germany, numbered as #1208, #1024, #1014, and #1020, respectively) were conjugated with bovine serum albumin (BSA) at a 1:2.5 ratio, as previously described [12]. C18[2]c solution was introduced to the agarose medium at a final concentration of 50 μm. Each agarose medium unit was cooled down to room temperature, and sealed with DMEM/F-12 medium (Gibco Life Technologies), supplemented with 10% fetal bovine serum, and 100 U/mL each of both penicillin and streptomycin (PAN Biotech GmbH). Tissue samples were incubated at 37 °C with 5% CO_2_ for 9 or 11 days. Afterward, the tissue blocks were meticulously retrieved, and preserved in a 4% formaldehyde solution (Otto Fischar GmbH) for 24 h.

### 2.4. Hematoxylin and Eosin (HE) Staining

After being dehydrated and embedded in paraffin, tissue blocks were cut into 5 μm sections using a SLIDE4003E microtome (pfm Medical, Cologne, Germany). The sections were then fixed onto adhesive microscope slides and deparaffinized, before being stained via an automated slide-staining station (Gemini, manufactured by Thermo Fisher Scientific, Waltham, MA, USA), as previously described [16]. After being stained in hematoxylin for 5–10 min, the slides were rinsed in warm water for 10 min, stained in 0.3% eosin for 5 min, rinsed again with tap water, and successively dehydrated in 70%, 96%, and 100% ethanol. Then, the slides were treated with xylene, and sealed with glass coverslips.

### 2.5. Immunofluorescence and DAPI Staining

Tissue slices were deparaffinized and heated in citrate buffer (pH 6.0) for 30 min. Then, tissue slices were cooled and left in distilled water. Before being stained, the slices were rinsed twice with 0.1% Tween 20 (9127.1, Carl Roth GmbH, Karlsruhe, Germany) in PBS. To ensure comparable results, respective sets of tissue samples were analyzed using each detection antibody simultaneously. For PAX7 and MyoD staining, the slides were permeabilized in 0.1% Triton X-100 (T8787, Sigma-Aldrich, Steinheim, Germany) for 10 min, and blocked with UltraCruz Blocking Reagent for 60 min. For CD80, CD163, CD11c, CD206, MARCO, and PTGER3 staining, the slides were directly blocked with 10% BSA in PBS for 60 min. The following antibodies were used: rabbit anti-human CD80 (ab134120, Abcam, Cambridge, UK) diluted 1:1000 in 3% BSA, mouse anti-human CD163 (ab156769, Abcam, Cambridge, UK) diluted 1:200 in 3% BSA, rabbit anti-human CD11c (ab52632, Abcam, Cambridge, UK) diluted 1:100 in 3% BSA, rabbit anti-human CD206 (PA5-101657, Thermo Fisher Scientific, Waltham, MA, USA) diluted 1:100 in 3% BSA, rabbit anti-human MARCO (PA5-64134, Thermo Fisher Scientific) diluted 1:100 in 3% BSA, rabbit anti-human PTGER3 (PA5-102057, Thermo Fisher Scientific) diluted 1:100 in 3% BSA, Pax-7 (sc-81975, Santa Cruz Biotechnology, Dallas, TX, USA) diluted 1:100 in UltraCruz Blocking Reagent, or mouse anti-human MyoD (sc-377460 AF488, Santa Cruz Biotechnology) 1:100 in UltraCruz Blocking Reagent (Santa Cruz Biotechnology). After the primary staining, the slices were then treated using the VectaFluor Amplified Kit containing anti-mouse IgG DyLigh 488 and 594 (DK2488 and 1594, Vector Laboratories, Burlingame, CA, USA). Next, the slices were treated with 0.1% DAPI (D9542, Sigma-Aldrich) in distilled water for 5 min. Then, the slides were sealed with glass coverslips in the Immu-Mount (9990402, Thermo Fisher Scientific).

### 2.6. Microscopy and Imaging

An automated microscope (DM6000B, Leica Microsystems, Wetzlar, Germany) was used for brightfield microscopy and the capturing of immunofluorescence (IF) images, with a 340–380 nm filter for DAPI; a 450–490 nm filter for PAX7, MYOD, or CD163; and a 590 nm filter for CD80, CD11c, CD206, MARCO, or PTGER3. Images were processed and merged via Diskus software (Leica). For evaluation, we captured overview IF images to locate potential positive cells. Finally, individual cells were further verified via zooming in, and different settings for the focus depth and exposure time. The average count of positive cells was obtained from nine microscopic fields in at least two random cross-sectional muscle fields of 0.24 mm^2^. Fields that contained adipose tissue, glands, or vessels were strictly excluded from the analysis.

### 2.7. Statistical Analyses

The Shapiro–Wilk test was utilized to assess the normal distribution of the variables. The correlation between two variables was determined using Pearson’s or Spearman’s rank correlation analysis. The relative fold changes in cell numbers by unsaturated or saturated FAs were compared using Wilcoxon tests or paired *t*-tests. GraphPad Prism (v8, GraphPad Software, San Diego, CA) was utilized for statistical analyses. The supplemented statistical analyses of the data are described in the description for the Supplementary Tables S1–S6. Differences with *p* values ≤ 0.05 were considered statistically significant.

## 3. Results

### 3.1. The Numbers of Macrophages and Stem Cells Correlated with the Expression of Inflammatory Proteins in Human Skeletal Muscle Tissue

**Figure 1:**
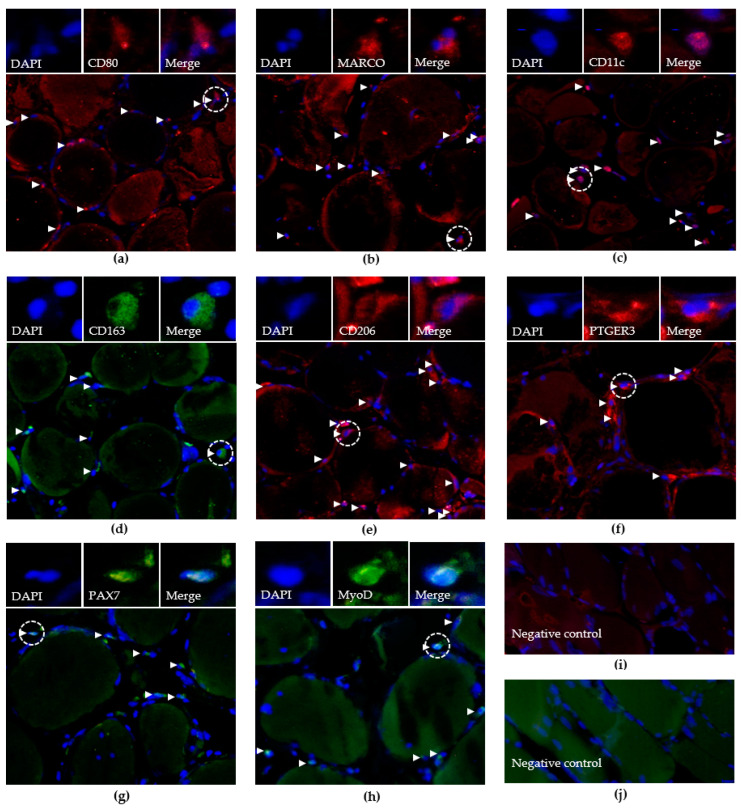
Representative IF images of the skeletal muscle tissue of a participant treated with U-FA for nine days. Each overview image corresponds to tissue fields of 270 µm × 210 µm. Tissue sections were stained using the DAPI and CD80 (**a**), MARCO (**b**), CD11c (**c**), CD163 (**d**), CD206 (**e**), PTGER3 (**f**), PAX7 (**g**), or MyoD (**h**) primary antibodies, or no primary antibodies as a negative control (**i**,**j**). Magnified smaller panels show images of a single positive cell (dashed line circle) using the DAPI (Blue, **left**) and Dylight 488 (Green, **middle**) or 594 (Red, **middle**) filters, or merged images (**right**). White arrows indicated the locations of positive cells, which were verified via advanced microscopy.

Understanding the crosstalk between muscle stem cells and macrophages is key to restoring muscle tissue regeneration. Current research on the regenerative processes in muscle tissue is largely based on animal experiments. Here, we used an in vitro model of human skeletal muscle tissue, to perceive the variety of tissue-resident stem cells and macrophages, and to study their response to different species of FA in the native environment of human skeletal muscle tissue. We included skeletal muscle tissue samples from 20 participants, who gave informed consent to donate their removed muscle tissue for research purposes after surgery. The clinical characteristics of the study participants and the skeletal muscle source of the tissue samples are summarized in Table 1. The participants were mostly older than 54 years. Two significantly younger participants (P7 and P19) were included, to examine possible age-related differences, as suggested previously [10,14,15,17]. The source and size of the skeletal muscle samples were strictly determined by the surgeons based on the aim and approach of the surgery. Most samples were obtained from the deep intrinsic back muscle (multifidus), followed by the abdominal muscle (obliquus externus abdominis), and limb muscles (vastus lateralis and pronator quadratus). Depending on the size of the donated tissue, we prepared multiple sections for the study of tissue-resident cells according to the distinct treatment and observation time. We prepared an untreated control section from all donors for reference analyses immediately after surgery, and subjected these sections to HE, DAPI, and immunofluorescence staining, using specific antibodies. Because human muscle-tissue-resident macrophages have not been systemically characterized before, we first tested a larger set of available antibodies against macrophage markers that were established in other human tissues. Antibodies against the macrophage markers CD11b, CD14, CD64, CD68, CD169, and TIMD4 revealed negligible signal intensities. This may be due to the poor quality of the antibodies, the low expression of the marker proteins, or the absence of positively expressing cells in muscle tissue. In the ultimate set of presented experiments, we utilized eight selected antibodies to exclusively detect CD80^+^, MARCO^+^, CD11c^+^, CD163^+^, CD206^+^ or PTGER3^+^, PAX7^+^, or MyoD^+^ cells. Tissue sections from 20 participants were embedded in paraffin, which is advantageous for the long-term storage of larger tissue sections, and the maintenance of tissue morphology. However, the maintained tissue morphology causes a stronger immunofluorescence (IF) background, compared to other techniques. For a better comparison between the participants, sections from all participants were simultaneously subjected to IF staining, using an individual antibody. The numbers of positive cells were determined using fluorescence microscopy in at least two randomly selected 0.24 mm^2^ cross-sectional fields of muscle tissue, each corresponding to nine macroscopic fields. Figure 1 provides a set of representative overview images obtained using CD80, MARCO, CD11c, CD163, CD206 or PTGER3, PAX7, or MyoD (Figure 1a–j). Indistinct signals in the overview were verified via further detailed macroscopy, repeatedly. In comparison, the total number of detected cell phenotypes strongly varied among the participants (Table 1). Further statistical analyses revealed no significant correlations between the number of CD80^+^, MARCO^+^, CD11c^+^, CD163^+^, CD206^+^, or PTGER3^+^ cells and the age (*p* > 0.11) or sex (*p* > 0.13) of the participants (Supplementary Tables S1 and S2). A weak correlation was detected between the body mass index (BMI) and the number of PTGER3^+^ macrophages (Supplementary Table S3, r = 0.58, *p* < 0.01). In contrast to the previous assumptions, the number of muscle stem cells in younger participants was not higher than that in older participant in our study (Table 1). In fact, we found moderate positive correlations between the donor age and the number of PAX7^+^ (r = 0.69, *p* < 0.001) and MyoD^+^ (r = 0.55, *p* < 0.01) stem cells (Supplementary Table S1). Therefore, we included the two younger donors in the further studies.

Next, we aimed to assess a possible link between the expression levels of inflammatory proteins and the numbers of detected macrophages or stem cells in muscle samples. The cytokine/chemokine expression profile in human muscle tissue had not been reported or studied before. We chose a multiplex approach for the detection of 34 human cytokines/chemokines, as listed in Materials and Methods. The preparation of muscle protein extracts for multiplex quantification required a minimum amount of 100 mg native muscle tissue. Six participants (P1, P2, P6, P12, P19, and P20) were excluded from this analysis, due to the limited sample size. Thus, an equal amount (2.5 mg tissue/sample) of tissue extract from 14 samples (P3–5, P7–11, P13–15, and P16–18) was prepared, and used for further analysis.

**Figure 2:**
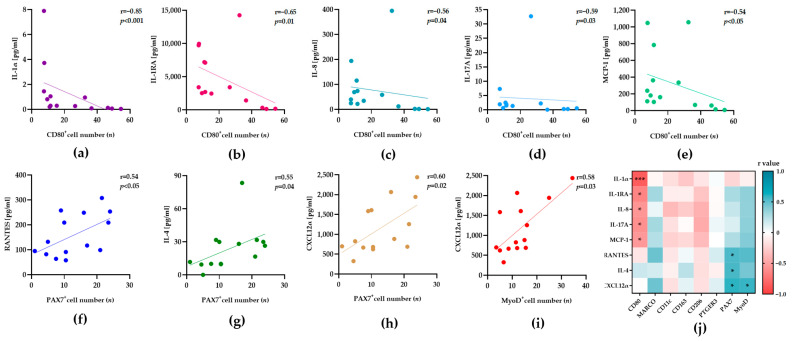
The numbers of macrophages and stem cells correlate with the expression of inflammatory proteins in human skeletal muscle tissue. Tissue samples (*n* = 14) were subjected to immunofluorescence staining for the detection of macrophages and stem cells. Aliquots of the corresponding samples were used to prepare extracts for cytokine/chemokine expression analysis. The concentration of 30 cytokines/chemokines was measured four times, to determine the mean values of the expression levels (Supplementary Table S4). Pearson correlation and Spearman’s rank correlation analyses were used to determine statistically significant relationships between the variables, cell numbers (*x*-axis), and cytokine/chemokine expression (*y*-axis) in the skeletal muscle tissue from 14 participants (*n* = 14). (**a**–**i**) Dot plots show the correlations between the number of individual cell phenotypes (*n*, y-axes) and the indicated levels of cytokines/chemokines (pg/mL, x-axes). The calculated correlation coefficient (r) and significance level (*p*) are presented in all plots. (**j**) The heatmap summarizes the direction (negative = red or green = positive), strength (r), and significance (*p*) of the calculated correlations between the number of individual cell phenotypes (**bottom**) and the levels of cytokines/chemokines (**left**) *p* ≤ 0.05 (*), *p* ≤ 0.001 (***).

The mean values of four independent measurements were used to determine the expression levels of 34 human cytokines/chemokines in each sample. In all samples, we found 30 cytokines/chemokines, expressed at very different levels (Supplementary Table S4). Again, the expression levels were not significantly different in the younger participant P7 (Supplementary Table S4). Four cytokines/chemokines, GM-CSF, MIP-1α, IL-10, and IFNγ, were not detectable in all samples. Pearson correlation and Spearman’s rank correlation analyses were performed to assess the relationships between the number of different cells and the expression level of all detected cytokines/chemokines. IL-1α, IL-1RA, IL-4, IL-6, IL-8, IL-15, IL-17A, IL-21, IL-22, MCP-1, RANTES, and CXCL12α revealed significant correlations in the entire study (Table 2). Although low in some samples, the expression levels of IL-1α (r = −0.85, *p* < 0.01), IL-1RA (r = −0.65, *p* = 0.01), IL-8 (r = −0.56, *p* = 0.04), IL-17A (r = −0.59, *p* = 0.03), and MCP-1 (r = −0.54, *p* < 0.05) were correlated with the number of CD80^+^ macrophages (Figure 2a–e). These correlations partially resembled the reported expression patterns of previously reported M1 macrophages.

The numbers of PAX7^+^ (r = 0.60, *p* = 0.02) and MyoD^+^ (r = 0.58, *p* = 0.03) stem cells were positively correlated with the expression level of CXCL12α, a recognized chemoattractant for muscle stem cells. This observation confirmed the previously suggested role of CXCL12α in the activation of primary human skeletal myoblasts in cell culture experiments [12]. Interestingly, we found two unexpected correlations of PAX7^+^ stem cells with the expression levels of the anti-inflammatory cytokine IL-4 (r = 0.55, *p* = 0.04), and the proinflammatory chemokine RANTES (r = 0.54, *p* = 0.04). Of note, some of the plots in Figure 2 exposed several outliers that correspond with different donors. The calculated correlation coefficients (r values, color scale), and their corresponding levels of significance (indicated by asterisks), are summarized in the heatmap of Figure 2j, for a better overview. The heatmap emphasizes the selective effect of CXCL12α on muscle stem cells, as its expression does not correlate with the numbers of the macrophage subtypes. Moreover, we assumed a positive effect of IL-4 and RANTES on tissue-resident stem cells, as their expression was not associated with any other macrophage subtypes.

### 3.2. The Phenotypes of Tissue-Resident Macrophages and Stem Cells Are Linked to Cytokine/Chemokine Expression in Human Skeletal Muscle Tissue

**Figure 3:**
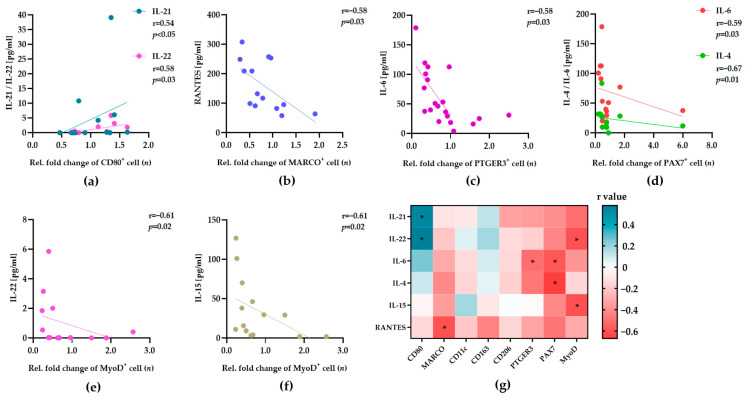
The levels of cytokines/chemokines affect the abundance of tissue-resident macrophages and stem cells in human skeletal muscle tissue. Tissue samples (*n* = 14) were subjected to immunofluorescence staining for the detection of macrophages and stem cells, before and after maintenance in vitro over 9 to 11 days. The number of cells in the tissue samples after maintenance in vitro was normalized to the corresponding cell numbers in the samples before maintenance in vitro, to obtain the relative fold change in cell numbers. Aliquots of the samples before maintenance in vitro were used to prepare extracts for the cytokine/chemokine expression analysis. Pearson correlation and Spearman’s rank correlation analyses were used to determine statistically significant relationships between the relative fold change in different cell numbers (*x*-axis) and cytokine expression (*y*-axis). (**a**–**f**) Dot plots show the correlations between the relative fold change in different cell phenotype numbers (*n*, y-axes) and the indicated levels of cytokines/chemokines (pg/mL, x-axes). The calculated correlation coefficient (r) and significance level (*p*) are presented in all plots. (**g**) The heatmap summarizes the direction (negative = red or green = positive), strength (r), and significance (*p*) of the calculated correlations between the number of individual cell phenotypes (**bottom**) and the levels of cytokines/chemokines (**left**) *p* ≤ 0.05 (*).

To analyze temporal changes in the resident cell counts, we maintained two sections of donor samples (*n* = 20) in vitro for 9 and 11 days, as described previously [16]. All samples were subjected to HE, DAPI, and immunofluorescence staining, to obtain the numbers of cells, as described above (Section 3.1). We generally observed a continuous change in the number of cells during the tissue maintenance in vitro. The numbers of macrophages were not different after 9 or 11 days, while the stem cells reached their highest numbers 11 days after surgery. To obtain a comparable relative fold change in the number of different cells, we normalized the maximum number of cells after 9 or 11 days to the cell numbers detected in the corresponding samples immediately after surgery (day 0). Thus, values lower that 1 indicate a decrease, and values higher than 1 indicate an increase in cell numbers, respectively (Figure 3a–f, x-axes). Overall, all donors showed different trends in the relative fold changes in macrophage and stem cell counts. However, we did not find significant correlations between the relative fold changes in cell phenotypes and donor characteristics (age, sex, BMI) or the sample source. Next, we sought to find possible relationships between the initial expression levels of cytokines/chemokines in the tissue and the relative fold changes in the cell phenotypes in vitro. Therefore, we performed a correlation analysis of the datasets from 14 samples that were subjected to multiplex protein analyses (Section 3.1). The Pearson correlation and Spearman’s rank correlation analyses revealed marked correlations between the expression of distinct cytokines/chemokines and the relative fold changes in the cell phenotypes (Figure 3a–f).

We found significant correlations between the relative numbers of CD80^+^, MARCO^+^, PTGER3^+^ PAX7^+^, and MyoD^+^ cells and the levels of different cytokines (Figure 3a–f). The relative fold changes in CD206^+^, CD163^+^, and CD11c^+^ macrophages were not correlated with the expression levels of 30 cytokines/chemokines. The relative fold changes in CD80^+^ macrophages were positively correlated with the expression levels of IL-21 and IL-22 (Figure 3a). In contrast, all other observed correlations followed a negative trend. The MARCO^+^, PTGER3^+^, PAX7^+^, and MyoD^+^ cells decreased with the higher expression of RANTES, IL-6 and IL-4, IL-22, or IL-15, respectively (Figure 3b–f). This may suggest the negative effect of RANTES, IL-6 and IL-4, IL-22, or IL-15 on the abundance of the corresponding cells. Of note, the limited size of the in vitro maintained tissue samples did not allow the preparation of tissue extracts for the detection of cytokines/chemokines. However, the expression levels of IL-21, IL-22, RANTES, IL-6, IL-4, and IL-15 most likely affect the phenotypes of macrophages and stem cells in human skeletal muscle tissue.

### 3.3. FAs Increase the Numbers of Macrophages and Stem Cells in Human Skeletal Muscle Tissue

**Figure 4:**
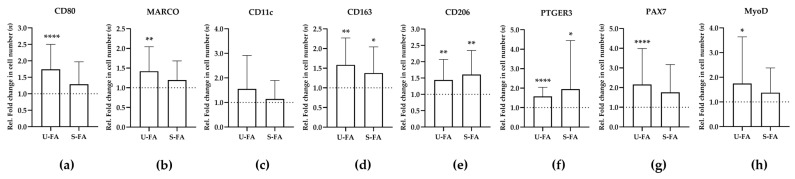
FAs promote distinct phenotypes of macrophages and stem cells in human skeletal muscle tissue. Skeletal muscle tissue sections from all participants (*n* = 20) were treated with U-FAs or S-FAs, or left untreated for 9 or 11 days. The number of cells in the FA-treated tissue samples was normalized to the corresponding cell numbers in the untreated tissue samples, to obtain the relative fold change in cell numbers (**a**–**h**). Thus, the cell numbers in untreated samples were set to 1 (dashed line). Diagrams show the relative fold changes in the number of cells (y-axes) of the indicated cell phenotype (top of each diagram) after treatment with U-FAs or S-FAs (x-axes). A paired *t*-test or Wilcoxon signed-rank test was utilized to assess the significance of the detected differences. The corresponding data are presented in Supplementary Table S5. *p* ≤ 0.05 (*), *p* ≤ 0.01 (**), *p* ≤ 0.0001 (****).

Animal studies have demonstrated that specific species of FAs can directly induce inflammatory gene expression in the skeletal muscle tissue of obese rats [18,19,20]. We assumed that FAs might activate tissue regeneration through a distinct set of stem cells and macrophages in the skeletal muscle tissue samples. We were able to examine this hypothesis in all participants. Five sections of each muscle tissue sample were individually maintained without, or with, four different FAs, saturated C16 and C18, or unsaturated C16[1]c and C18[2]c, for 9 or 11 days in vitro. The nomenclature of the FAs was simplified according to the number of carbon atoms, which is depicted first, followed by square brackets enclosing the number of unsaturated carbon double bonds, and the letter “c” signifying the cis or trans configuration. Firstly, different phenotypes of macrophages and stem cells were determined in all samples via HE, DAPI, and immunofluorescence staining. The corresponding sets of five tissue sections per participant were analyzed simultaneously. Secondly, the cell counts were compared between the FA-treated samples and the untreated control, to obtain the relative fold change in the cell counts after treatment. Overall, we found almost identical effects elicited by the saturated FAs (S-Fas) C16 and C18, or by the unsaturated fatty acids (U-FAs) C16[1]c and C18[2]c. The data showed that only U-FAs were able to significantly increase the CD80^+^ and MARCO^+^ macrophages, which have been previously classified as M1 macrophage markers (Figure 4a–c, and Supplementary Table S5). Further statistical analysis revealed no significant differences between the effects of US-FA or S-FA on the number of CD80^+^, MARCO^+^, CD11c^+^, CD163^+^, CD206^+^, or PTGER3^+^ cells in male or female participants (Supplementary Table S6). Only the insignificant effect of S-FA on PAX7^+^ cells was slightly different between male and female participants. In contrast, CD163^+^, CD206^+^, and PTGER3^+^ macrophages were significantly increased by both saturated and unsaturated FAs (Figure 4d–f). Importantly, PAX7^+^ and MyoD^+^ stem cells were specifically increased by unsaturated FAs, in accordance with CD80^+^ and MARCO^+^ macrophages (Figure 3a–c). These results agree with the beneficial role of unsaturated FAs on muscle stem cells.

### 3.4. Saturated and Unsaturated FAs Induce a Coordinated Increase in Different Macrophages and MyoD^+^ Stem Cells

**Figure 5:**
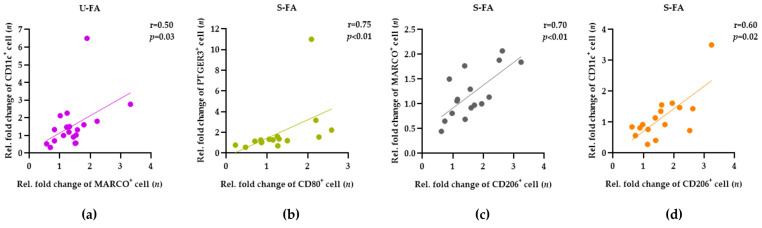
The FA-mediated responses of different macrophage phenotypes are correlated in human skeletal muscle tissue. The relative fold change in the numbers of the macrophage phenotypes was determined, as described in the legend of Figure 4. Pearson correlation and Spearman’s rank correlation analyses were used to determine the relationships between the fold changes in the macrophage phenotypes in the skeletal muscle tissue of all participants (*n* = 20). (**a**–**d**) Dot plots present the strength (r) and the significance (*p*) of the correlations between the fold change in the numbers of the macrophage phenotypes (x and y-axes) after treatment with U-FAs and S-FAs, as indicated at the top of each plot.

The uniform response of muscle-tissue-resident macrophages to FAs raised the question of whether the activation of distinct macrophage subtypes may be correlated. Therefore, we performed Pearson correlation and Spearman’s rank correlation analyses, to assess a possible link between the response of different macrophage phenotypes to FAs in tissue samples from all participants (*n* = 20). As expected, no correlations were found between the FA responses of MyoD^+^ and PAX7^+^ stem cells or between stem cells and macrophages. This highlighted the functional diversity of stem cells and macrophages. In samples treated with unsaturated FAs, we found a significant correlation between the responses of CD11c^+^ and MARCO^+^ macrophages (Figure 5a). In samples treated with saturated FAs, we detected a strong and significant correlation between the CD80^+^ and PTGER3^+^ phenotypes (Figure 5b), CD206^+^ and MARCO^+^ (Figure 5c), or CD206^+^ and CD11c^+^ (Figure 5d) which were significantly high between 0.6 and 0.75. Taken together, our data suggest that the coordinated response of muscle-tissue-resident macrophages is superior to the proposed dichotomy for M1/M2 macrophage activation.

## 4. Discussion

The age-related decline of skeletal muscle mass and strength is a determining factor in the loss of agility and independence, ultimately leading to morbidity and mortality [21]. The number of individuals aged over 65 years will reach 1.5 billion in 2050, according to the World Health Organization. The progress of population aging makes skeletal muscle wasting a major challenge to health organizations worldwide [22]. Therefore, understanding and targeting the mechanisms of muscle regeneration is of tremendous value to the development of new therapeutic strategies. Our study was the first attempt to investigate the early phases of regenerative processes in a native human skeletal muscle model that allows for prolonged experimental observations. The outcomes demonstrated the persisting capability of stem cells in young and aging muscle tissue of responding to macrophages and metabolic signals, such as FAs. Importantly, this report substantiated the capability of the human skeletal muscle tissue model of testing new compounds and reducing animal experiments.

Some limitations in our study model, however, must be noted. Firstly, our study model allows for the static, rather than the dynamic, assessment of human skeletal muscle tissue samples over time. While this limitation generally applies to all histological studies, the data presented here signify the changes in cell abundance or phenotypes only over the empirically defined time intervals of 0, 9, or 11 days. In preliminary experiments, however, we also maintained parallel samples for 5, 7, and 9 days, and observed equal trends in all samples from the same tissue. Thus, the indicated relative fold change in the frequency of the depicted phenotype represents the trend over 11 days. Secondly, the histological assessments in our study relied on a set of previously established cell markers, and the quality of the available detection antibodies. In preliminary experiments, we tested the available antibodies for the detection of many cell markers that were associated with skeletal muscle stem cell or tissue-resident macrophages. Despite many efforts, none of the antibodies revealed detectable or reliable signals. Accordingly, many potentially relevant but unidentified cell phenotypes remain to be characterized in the human skeletal muscle samples used in the current study.

Human studies of skeletal muscle tissue in older adults are rare, because of the restricted access to eligible donors and to adequate quantities of specimens. Alternatively, rodent models have been exploited in the study of muscle tissue regeneration in aging and disease, assuming that the number of skeletal muscle stem cells decreases with age, while the number of M1 macrophages in muscle tissue increases with age and obesity [23,24,25]. However, none of these assumptions corresponded to the donors’ characteristics in our study, which included seven obese individuals, and two lean younger adults. For instance, we found the highest number of stem cells in an obese 82-year-old participant (P16). Moreover, neither the number nor the FA response of the macrophages and stem cells was significantly different in the two younger participants (P7 and P19). Therefore, we suggest that the results obtained from animal models with identical genetic backgrounds under similar physiological conditions cannot translate to the characteristics of native human muscle tissue. Indeed, animal models convey different sets of macrophages and cell markers, and lack equivalent genes to the relevant cytokines/chemokines in humans. Both biological and environmental factors most likely influence the development, abundance, and fate of human skeletal-muscle-tissue-resident stem cells and macrophages.

Three cytokines/chemokines, namely, CXCL12α, IL-4, and RANTES, were identified in the current study as playing a significant role in the abundance of muscle stem cells. Previously, CXCL12α was shown to activate STAT3 signaling, which inhibits the expression of PAX7 and MyoD in human skeletal muscle stem cells developed from bone-marrow-derived stem cells in vitro [26]. Thus, a possible mechanism of anti-myogenic action was hypothesized for CXCL12α [27]. Additionally, our recent clinical study of 80 older adults revealed that higher CXCL12α plasma levels were significantly correlated with the loss of muscle mass and strength. These findings are interesting, because they seem to contradict the positive correlation between CXCL12α tissue expression and the number of stem cells, in the current study. However, the loss of muscle mass was correlated with elevated CXCL12α in the plasma, and not in the skeletal muscle tissue. In addition, the total elevation in plasma CXCL12α might not truly represent the effective level of CXCL12α in the skeletal muscle tissue, which drives the local abundance of PAX7^+^ and MyoD^+^ muscle stem cells. A possible role of IL-4 and RANTES in the regulation of muscle stem cells in human skeletal muscle tissue has not yet been reported, and remains to be clarified.

The outcomes from the human muscle tissue model correspond to the pivotal role of macrophages in tissue regeneration, although the analysis of tissue-resident macrophages in many human organs remains limited. The levels of four proinflammatory chemokines, IL-1α, IL-8, IL-17A, and IL-1RA, correlated exclusively with the number of tissue-resident CD80^+^ macrophages. Moreover, the increase in CD80^+^ macrophages was associated with the elevated expression of two additional proinflammatory proteins, IL-21 and IL-22, in human muscle tissue. These findings agree with the classification of CD80^+^ macrophages as proinflammatory M1 macrophages. However, the number of CD80^+^ macrophages was inversely correlated with another proinflammatory chemokine, MCP-1, which is known to attract other inflammatory cells [28]. Thus, it is conceivable that the profile and function of muscle-tissue-resident macrophages markedly differ from the classic definition of bone-marrow-derived M1/M2 macrophages. Tissue-resident macrophages have long been recognized as regeneration rheostats in many different tissues and species; however, it is still unclear which phenotypes are involved in this process [29]. A large spectrum of activation pathways were identified that lead to different profiles of tissue macrophages [30]. Thus, caution is recommended regarding the general classification of tissue-resident macrophages into M1/M2 nomenclature without further investigation of different tissues and species [6].

Recent studies have reported a difference in the expression profiles of monocyte-derived macrophages between males and females [31]. Despite the limited number of participants, a further statistical analysis of male and female participants revealed no significant differences between the number of CD80^+^, MARCO^+^, CD11c^+^, CD163^+^, CD206^+^, PTGER3^+^, or MyoD^+^ cells and their responses to U-FA and S-FA treatments (Supplementary Table S6). Similarly, no sex-related differences were found in the number of PAX7^+^ cells, without or with U-FA treatment. Although S-FA did not affect the PAX7^+^ cells, we note a moderate level of significance via the comparison of the S-FA response of PAX7^+^ cells between male and female participants (Supplementary Table S6). Nevertheless, the study of sex-related differences could be more relevant in a larger group of participants.

An essential component of our study model is the embedding material that imparts elastic consistency, and allows the free diffusion of small molecules, such as FAs, into the embedded material. As expected, the unsaturated and saturated FAs significantly affected the number of macrophages and stem cells. CD80^+^ and MARCO^+^ cells, and PAX7^+^ and MyoD^+^ stem cells were increased by unsaturated FA, explicitly, and to an almost equal extent. To our knowledge, this is the first experimental demonstration of an activation link between resident CD80^+^ and MARCO^+^ macrophages and stem cells in human skeletal muscle tissue. Although not excluded by our data, possible links between stem cells and CD163^+^, CD206^+^, and PTGER3^+^ macrophages seem unlikely. Firstly, M2 phenotypes showed a uniform response to the unsaturated and saturated FAs. Secondly, the saturated FAs did not affect the number of PAX7^+^ and MyoD^+^ stem cells. From a different perspective, however, our data endorsed a coordinated increase in the explicit macrophage phenotypes caused by the saturated FAs. We found strong cross correlations between the M1 and M2 phenotypes in response to the FAs. It is tempting to speculate that saturated FAs impart two different paths of coordinated actions in human skeletal muscle tissue, between different macrophage phenotypes, and between stem cells and CD80^+^ and MARCO^+^ macrophages.

## 5. Conclusions

The process of skeletal muscle regeneration is complex, involving numerous types of cells, signaling molecules, and physiochemical components. Various animal models have been employed to develop new strategies for improving skeletal muscle regeneration in older adults. However, there is still a high demand for human study models to bring research approaches closer to clinically applicable therapies. Our study presents the first attempt to investigate the proposed beneficial effect of FAs on the interactions of resident macrophages and stem cells in native human skeletal muscle tissue. Unsaturated long-chain FAs specifically induce the coordinated activation of muscle stem cells, and distinct macrophage phenotypes that are classified as proinflammatory. Hence, the human skeletal muscle model provides a versatile tool for the future testing of other compounds, nutrition, or stimuli. Furthermore, the development of current technologies, such as single-cell and spatial transcriptomics, can tremendously expand our knowledge of stem cell and macrophage biology.

## Figures and Tables

**Table 1:** Participant characteristics and the number of positive cells in 0.24 mm^2^ of muscle tissue.

Participant	Sex	Age (Years)	BMI (kg/m^2^)	T2D	Sample Source	M1 Macrophage Markers			M2 Macrophage Markers			Stem Cells	
						CD80^+^ (*n*)	MARCO^+^ (*n*)	CD11c^+^ (*n*)	CD163^+^ (*n*)	CD206^+^ (*n*)	PTGER3^+^ (*n*)	PAX7^+^ (*n*)	MyoD^+^ (*n*)
P1	F	79	22.8	No	1	26.0	24.5	19.0	27.0	26.7	18.5	22.5	18.0
P2	F	67	46.9	No	3	19.0	19.0	32.0	9.5	22.5	34.0	23.5	14.0
P3	M	71	35.1	Yes	1	36.5	25.5	47.0	40.5	60.5	24.0	17.0	15.0
P4	F	69	24.8	No	1	46.5	12.0	19.3	18.3	35.0	20.7	10.5	5.0
P5	F	55	16.7	No	2	7.5	11.0	17.5	12.0	13.5	12.0	7.5	8.5
P6	F	64	33.8	No	2	24.0	21.5	8.5	7.0	31.5	21.5	6.0	15.0
P7	M	15	17.3	No	2	11.5	23.5	76.5	7.0	30.0	1.5	1.0	3.5
P8	M	75	30.5	No	4	7.7	32.3	17.0	14.5	17.0	19.5	21.5	16.0
P9	F	60	22.1	No	2	15.3	20.3	20.5	8.0	19.7	20.0	9.0	5.0
P10	F	57	22.3	No	4	11.0	20.0	12.0	11.0	14.3	10.0	10.0	13.5
P11	M	54	40.1	No	4	7.5	30.0	62.0	13.0	64.0	26.5	5.0	11.5
P12	F	55	22.6	No	2	3.5	16.0	36.5	1.5	37.5	9.0	2.0	2.5
P13	F	69	22.3	No	2	32.5	32.5	67.5	10.0	17.5	16.0	23.5	25.0
P14	M	66	26.8	No	3	9.5	18.0	21.5	6.5	23.0	24.5	4.5	6.5
P15	F	70	20.0	No	2	11.5	34.0	32.0	9.0	55.0	14.0	16.0	12.0
P16	F	82	30.5	No	1	32.5	49.0	138.5	54.0	142.0	46.0	87.0	90.5
P17	M	66	33.6	No	1	49.0	39.5	82.5	9.5	23.0	10.0	10.5	12.0
P18	M	67	28.6	No	2	26.5	36.0	33.7	22.5	15.0	29.5	24.0	34.5
P19	F	22	20.3	No	2	54.5	28.5	34.0	25.0	52.0	23.5	21.0	15.5
P20	M	65	24.2	No	2	39.5	45.0	25.5	27.5	25.0	12.0	22.0	21.5

T2D—type 2 diabetes; F—female; M—male; 1—vastus lateralis muscle; 2—multifidus muscle; 3—pronator quadratus muscle; 4—obliquus externus abdominis muscle.

**Table 2:** The levels of cytokines/chemokines in participants’ skeletal muscle tissue as the mean of four measurements by a coefficient of variations (CV) < 5%.

Participants	IL-1α (pg/mL)	IL-1RA (pg/mL)	IL-4 (pg/mL)	IL-6 (pg/mL)	IL-8 (pg/mL)	IL-15 (pg/mL)	IL-17A (pg/mL)	IL-21 (pg/mL)	MCP-1 (pg/mL)	RANTES (pg/mL)	IL-22 (pg/mL)	CXCL12α (pg/mL)
P3	0.09	1419.09	83.4	178.71	12.15	15.69	0.03	10.83	68.61	117.3	0.03	883.71
P4	0.13	324.55	9.98	39.95	2.2	29.15	0.27	0.01	61.38	57.91	0.01	624.34
P5	1.45	3408.88	9.98	36.38	24.77	1.87	1.94	0.01	112.63	63.7	0.01	667.18
P7	0.3	2693.18	11.75	37.59	22.32	1.67	1.49	0.15	104.22	95.2	0.42	698.87
P8	3.72	9960.9	31.68	100.71	194.31	9.36	1.98	4.23	1047.99	307.95	2.01	1257.9
P9	0.3	2461.8	31.68	112.77	34.35	70.05	1.35	39.09	161.73	257.76	5.85	1584.45
P10	0.21	7195.47	29.91	112.83	115.35	101.01	2.52	6.09	362.55	209.07	3.15	1609.02
P11	7.89	9729.93	0	50.88	39.99	38.04	7.32	0.03	238.44	132.21	0.03	826.77
P13	0.96	14,250.84	29.94	90.84	394.74	126.96	2.22	0.03	1056.24	209.31	0.54	1941.87
P14	0.82	2522.2	9.39	29.43	69.27	11.02	0.64	0.19	181.34	82.26	1.85	326.47
P15	1.05	7109.25	28.17	77.1	73.92	29.55	1.92	0.03	784.26	248.7	0.03	2066.04
P16	0.06	1718.93	10.57	25.28	25.97	5.45	0.84	0.01	67.65	91.89	0.01	624.59
P17	0.07	136.88	9.68	20.26	1.88	3.32	0.27	0.01	16.11	91.31	0.01	682.36
P18	0.27	3412.05	26.43	53.19	58.47	46.35	32.7	0.03	335.46	253.5	0.03	2437.89

## Data Availability

The data presented in this study are available in Supplementary Materials.

## Supplementary Materials

The following supporting information can be downloaded at: https://www.mdpi.com/article/10.3390/biology12081111/s1, Table S1: Spearman’s test of the variables, age, and cell numbers on day 0.; Table S2: Unpaired *t*-test and Mann–Whitney test of variables, sex, and cell numbers on day 0.; Table S3: Pearson test and Spearman’s test of variables, BMI, and cell numbers on day 0.; Table S4: Mean expression levels of thirty cytokines/chemokines (*p*g) in human skeletal muscle tissue extracts (ml).; Table S5: Average fold change in cell numbers by FA stimulation.; Table S6: Unpaired *t*-test and Mann–Whitney test of differences between male and female participants in the number of cells (untreated) or the relative fold change in cell numbers in response to unsaturated (U-FA) or saturated fatty acids (S-FA).
