# Supplementary material for: Unsaturated Long-Chain Fatty Acids Activate Resident Macrophages and Stem Cells in a Human Skeletal Muscle Tissue Model
Source: Biology (Basel). 2023 Aug 9;12(8):1111. doi: 10.3390/biology12081111 (PMC10452335; doi:10.3390/biology12081111)
Supplement: Supplementary file 1 [file biology-12-01111-s001.zip › biology-2502564-supplementary.pdf]

Supplementary Table S1: Spearman's correlation analysis of the variables, age, and cell numbers on day 0. Significant correlations are marked in green.

| | CD80 | MARCO | CD11c | CD163 | CD206 | PTGER3 | PAX7 | MyoD |
|---|---|---|---|---|---|---|---|---|
| r | 0.2993 | 0.3652 | -0.0177 | 0.4461 | 0.0814 | 0.3549 | 0.6888 | 0.5518 |
| *p* | 0.1999 | 0.1133 | 0.9410 | 0.0487 | 0.7331 | 0.1246 | 0.0008 | 0.0117 |

Supplementary Table S2: Unpaired t test and Mann Whitney U test of variables, sex, and cell numbers on day 0.

| | CD80 | MARCO | CD11c | CD163 | CD206 | PTGER3 | PAX7 | MyoD |
|---|---|---|---|---|---|---|---|---|
| *p* | 0.9799 | 0.1328 | 0.2302 | 0.6645 | 0.9547 | 0.6738 | 0.6372 | 0.8950 |

Supplementary Table S3: Pearson's correlation analysis and Spearman's correlation analysis of variables, BMI, and cell numbers on day 0. Significant differences are marked in green.

| | CD80 | MARCO | CD11c | CD163 | CD206 | PTGER3 | PAX7 | MyoD |
|---|---|---|---|---|---|---|---|---|
| r | 0.0680 | 0.1592 | 0.1212 | 0.2041 | 0.2326 | 0.5831 | 0.2383 | 0.2825 |
| *p* | 0.7757 | 0.5027 | 0.6108 | 0.3881 | 0.3237 | 0.0070 | 0.3116 | 0.2275 |

Supplementary Table S4: Expression levels of thirty cytokines/chemokines (pg) in human skeletal muscle tissue extracts (ml). Mean values of four reads are presented by a coefficient of variations (CV) <5%. Twelve cytokines/chemokines (green fields) were correlated with different cell phenotypes as shown in Table 2, figure 1, and 2 of the manuscript.

| Participants | IL-1α | IL-1RA | IL-4 | IL-6 | IL-8 | IL-15 | IL-17A | IL-21 | MCP-1 | RANTES | IL-22 | CXCL12α |
|---|---|---|---|---|---|---|---|---|---|---|---|---|
| **P3** | 0.09 | 1419.09 | 83.4 | 178.71 | 12.15 | 15.69 | 0.03 | 10.83 | 68.61 | 117.3 | 0.03 | 883.71 |
| **P4** | 0.13 | 324.55 | 9.98 | 39.95 | 2.2 | 29.15 | 0.27 | 0.01 | 61.38 | 57.91 | 0.01 | 624.34 |
| **P5** | 1.45 | 3408.88 | 9.98 | 36.38 | 24.77 | 1.87 | 1.94 | 0.01 | 112.63 | 63.7 | 0.01 | 667.18 |
| **P7** | 0.3 | 2693.18 | 11.75 | 37.59 | 22.32 | 1.67 | 1.49 | 0.15 | 104.22 | 95.2 | 0.42 | 698.87 |
| **P8** | 3.72 | 9960.9 | 31.68 | 100.71 | 194.31 | 9.36 | 1.98 | 4.23 | 1047.99 | 307.95 | 2.01 | 1257.9 |
| **P9** | 0.3 | 2461.8 | 31.68 | 112.77 | 34.35 | 70.05 | 1.35 | 39.09 | 161.73 | 257.76 | 5.85 | 1584.45 |
| **P10** | 0.21 | 7195.47 | 29.91 | 112.83 | 115.35 | 101.01 | 2.52 | 6.09 | 362.55 | 209.07 | 3.15 | 1609.02 |
| **P11** | 7.89 | 9729.93 | 0 | 50.88 | 39.99 | 38.04 | 7.32 | 0.03 | 238.44 | 132.21 | 0.03 | 826.77 |
| **P13** | 0.96 | 14250.84 | 29.94 | 90.84 | 394.74 | 126.96 | 2.22 | 0.03 | 1056.24 | 209.31 | 0.54 | 1941.87 |
| **P14** | 0.82 | 2522.2 | 9.39 | 29.43 | 69.27 | 11.02 | 0.64 | 0.19 | 181.34 | 82.26 | 1.85 | 326.47 |
| **P15** | 1.05 | 7109.25 | 28.17 | 77.1 | 73.92 | 29.55 | 1.92 | 0.03 | 784.26 | 248.7 | 0.03 | 2066.04 |
| **P16** | 0.06 | 1718.93 | 10.57 | 25.28 | 25.97 | 5.45 | 0.84 | 0.01 | 67.65 | 91.89 | 0.01 | 624.59 |
| **P17** | 0.07 | 136.88 | 9.68 | 20.26 | 1.88 | 3.32 | 0.27 | 0.01 | 16.11 | 91.31 | 0.01 | 682.36 |
| **P18** | 0.27 | 3412.05 | 26.43 | 53.19 | 58.47 | 46.35 | 32.7 | 0.03 | 335.46 | 253.5 | 0.03 | 2437.89 |

| Participants | Eotaxin | GRO-α | IFN α | MIP-1 α | TNF-β | IL-1β | IL-2 | IL-5 | IL-7 | IL-9 | IL-13 | IL-18 | IL-23 | IL-27 | IL-31 | IP-10 | MIP-1β | TNF-α |
|---|---|---|---|---|---|---|---|---|---|---|---|---|---|---|---|---|---|---|
| **P3** | 3 | 13.8 | 0.99 | 3.6 | 0.03 | 0.03 | 0 | 0.03 | 16.86 | 1.65 | 7.92 | 233.49 | 0.03 | 0.03 | 78.45 | 22.14 | 94.32 | 9.36 |
| **P4** | 4.02 | 1.87 | 0.21 | 0.32 | 0.01 | 0.1 | 0.01 | 0.01 | 2.13 | 0.01 | 0 | 12.83 | 0.01 | 0 | 0.01 | 20.77 | 36.52 | 0.04 |
| **P5** | 10.98 | 45.07 | 0.42 | 2.27 | 1 | 0.83 | 0.01 | 0.01 | 5 | 0.39 | 1.59 | 70.66 | 0.01 | 5.51 | 0.01 | 17.21 | 202.73 | 1.33 |
| **P7** | 7.98 | 68.71 | 0.36 | 7.62 | 0.87 | 3.05 | 8.42 | 133.63 | 5.45 | 0.92 | 3.14 | 27.86 | 17.92 | 20.54 | 0.43 | 86.42 | 196.64 | 1.83 |
| **P8** | 15.27 | 241.26 | 3.75 | 17.61 | 0.03 | 32.37 | 37.26 | 0.03 | 13.47 | 3.36 | 0 | 23.64 | 0.03 | 0.03 | 38.58 | 50.97 | 254.19 | 29.13 |
| **P9** | 10.95 | 105.45 | 3.87 | 3.27 | 17.91 | 2.79 | 0.03 | 0.03 | 14.31 | 3.45 | 0 | 36.24 | 16.38 | 0.03 | 8.52 | 55.8 | 359.76 | 8.52 |
| **P10** | 9.6 | 183.39 | 0.81 | 6.33 | 0.03 | 32.94 | 0.03 | 0.03 | 17.34 | 3.45 | 4.77 | 102.21 | 19.38 | 6.09 | 11.97 | 36.66 | 483.78 | 4.8 |
| **P11** | 17.94 | 76.59 | 2.91 | 4.02 | 0.03 | 2.22 | 0.03 | 0.03 | 10.71 | 6.27 | 0 | 26.4 | 0.03 | 121.65 | 0.03 | 28.89 | 116.67 | 26.58 |
| **P13** | 21.09 | 206.7 | 2.13 | 54.27 | 1.83 | 23.7 | 15.63 | 0.03 | 9.87 | 7.11 | 7.92 | 174.3 | 0.03 | 6.09 | 0.06 | 96.12 | 836.37 | 9.81 |
| **P14** | 3.21 | 30.43 | 0.52 | 7.41 | 0.01 | 3.86 | 5.21 | 15.81 | 5.22 | 0.92 | 0 | 32.48 | 0.01 | 0.01 | 9.94 | 18.18 | 100.91 | 2.1 |
| **P15** | 21.57 | 123.93 | 2.25 | 6.21 | 0.03 | 18.84 | 15.63 | 0.03 | 13.29 | 3.45 | 6.36 | 99.39 | 0.03 | 6.09 | 1.65 | 75.33 | 501.27 | 7.11 |
| **P16** | 3.51 | 11.86 | 0.42 | 0.87 | 0.01 | 0.83 | 0.01 | 41.81 | 3.17 | 0.87 | 1.59 | 16.91 | 0.01 | 5.51 | 0 | 13.72 | 93.17 | 1.33 |
| **P17** | 1.85 | 4.8 | 0.29 | 0.71 | 1.58 | 0.93 | 0.01 | 0.01 | 3.63 | 0.01 | 0 | 2.86 | 0.01 | 0.01 | 2.84 | 5.29 | 56.01 | 0.53 |
| **P18** | 52.77 | 105.48 | 2.13 | 4.5 | 0.03 | 6 | 60.75 | 22.35 | 10.89 | 13.65 | 6.36 | 57.48 | 0.03 | 70.86 | 2.91 | 89.4 | 366.21 | 11.79 |

Supplementary Table S5: Average fold change of cell numbers by FA stimulation. Data points of the diagrams of figure 3a-h are shown.

| | | Average fold change of cell number | Standard deviation | *P* value |
|---|---|---|---|---|
| CD80 | U-FA | 1.74 | 0.74 | <0.0001 |
| | S-FA | 1.29 | 0.66 | 0.1077 |
| MARCO | U-FA | 1.42 | 0.61 | 0.0033 |
| | S-FA | 1.20 | 0.47 | 0.1288 |
| CD11c | U-FA | 1.55 | 1.32 | 0.0737 |
| | S-FA | 1.15 | 0.73 | 0.7916 |
| CD163 | U-FA | 1.58 | 0.67 | 0.0016 |
| | S-FA | 1.38 | 0.65 | 0.0161 |
| CD206 | U-FA | 1.44 | 0.61 | 0.0047 |
| | S-FA | 1.61 | 0.71 | 0.0050 |
| PTGER3 | U-FA | 1.58 | 0.45 | <0.0001 |
| | S-FA | 1.95 | 2.41 | 0.0214 |
| PAX7 | U-FA | 2.16 | 1.78 | <0.0001 |
| | S-FA | 1.76 | 1.36 | 0.1072 |
| MyoD | U-FA | 1.75 | 1.84 | 0.0459 |
| | S-FA | 1.38 | 0.97 | 0.2293 |

Supplementary Table S6: Unpaired t test and Mann Whitney U test of differences between male and female participants in the number of cells (untreated) or the relative fold change in cell numbers in response to unsaturated (U-FA) or saturated fatty acids (S-FA) on day 9. Significant differences are marked in green.

| | | **CD80** | **MARCO** | **CD11c** | **CD163** | **CD206** | **PTGER3** | **PAX7** | **MyoD** |
|---|---|---|---|---|---|---|---|---|---|
| *p* | **untreated** | 0.3431 | 0.8129 | 0.1349 | 0.4849 | 0.8888 | 0.4269 | 0.6784 | 0.2304 |
| | **U-FA** | 0.4784 | 0.5448 | 0.4920 | 0.9000 | 0.1549 | 0.9678 | 0.4920 | 0.8852 |
| | **S-FA** | 0.1679 | 0.4727 | 0.2295 | 0.0586 | 0.2529 | 0.0727 | 0.0245 | 0.0727 |
